# Antibiotics Prescription Over Three Years in a French Benchmarking Network of 23 Level 3 Neonatal Wards

**DOI:** 10.3389/fphar.2020.585018

**Published:** 2021-01-25

**Authors:** Séverine Martin-Mons, Simon Lorrain, Silvia Iacobelli, Béatrice Gouyon, Jean-Bernard Gouyon, On behalf of the B-PEN Study Group

**Affiliations:** ^1^ Centre D’Etudes Périnatales de L’Océan Indien (Université de La Réunion), CHU de La Réunion Site Sud, Saint-Pierre, France; ^2^ Société LogipremF, Saint-Pierre, France

**Keywords:** neonatal intensive care unit, antibiotics, preterm, neonatal ward, newborn, CPOE, benchmarking

## Abstract

**Introduction:** Prescribing antibiotics to newborns is challenging, as excess antibiotics are a risk factor for increased morbidity and mortality. The objective of this study was to describe the evolution of antibiotic exposure over three years in a large network of level 3 neonatal wards where each center is informed yearly of its own results and the results of other centers and has full autonomy to improve its performance.

**Patients and Methods:** This is a prospective, observational study of antibiotics prescriptions over the 2017–2019 period in a network of 23 French level 3 neonatal wards. The network relied on an internal benchmarking program based on a computerized prescription ordering system. Among others, antibiotics exposure, treatment duration, and antibiotics spectrum index were analyzed.

**Results:** The population consisted of 39,971 neonates (51.5% preterm), 44.3% of which were treated with antibiotics. Of the treated patients, 78.5% started their first antibiotic treatment in the first three days of life. Antibiotic exposure rate significantly declined from 2017 to 2019 (from 46.8% to 42.8%, *p* < 0.0001); this decline was significant in groups with gestational age >26 weeks, but not in the group with extremely low gestational age <27 weeks. Gentamicin, cefotaxime, amoxicillin (ampicillin), vancomycin, and amikacin were the antibiotics most prescribed. The lower the gestational age, the higher the exposure for cefotaxime, vancomycin, and amikacin. Compared to 2017, cefotaxime exposure in 2019 declined by 12.6%, but the change was only significant in the gestational age group of 32–36 weeks (17.4%) and at term (20.3%). The triple combination of antibiotics in the first three days decreased by 28.8% from 2017 to 2019, and this was significant in each gestational age group. During the study, the delayed ending of antibiotics in unconfirmed early-onset neonatal infection increased from 9.6% to 11.9%.

**Conclusion:** This study showed that a strategy characterized by the collection of information via a computerized order-entry system, analysis of the results by a steering committee representative of all neonatal wards, and complete autonomy of neonatal wards in the choice of prescription modalities, is associated with a significant reduction in the use of antibiotics in newborns with gestational age greater than 26 weeks.

## Introduction

Prescription of antibiotics in neonatal intensive care units (NICUs) poses a unique challenge as prescription of antibiotics in excess is a risk factor for late-onset neonatal sepsis, abnormal digestive microbiota, necrotizing enterocolitis, candidemia, retinopathy of prematurity, bronchopulmonary dysplasia, and neonatal death (Alexander et al., 2011; Cotten, 2016; Esaiassen et al., 2018; Fjalstad et al., 2018; Firestein et al., 2019; Ting et al., 2019). Antibiotics overuse in neonatal wards (NWs) originates from the fear of overwhelming sepsis in an untreated infected baby, the lack of specific clinical and biological signs of neonatal infection, and the difficulty in obtaining efficient routine bacteriological examinations. These factors explain why up to 72% of neonates admitted to NICUs are given antibiotics and why this rate is the highest for the most immature babies, i.e., close to 100% in neonates with extremely low birth weight (ELBW, <1,000 g) or extremely low gestational age (ELGA, <27 weeks) (Gouyon et al., 2019). These high rates are markedly in contrast with the scarcity of proven early-onset neonatal infection (EONI), which has been rated as low as 2.5% in symptomatic ELBW infants in the United States (Puopolo et al., 2017) and at 2.3% in the United Kingdom (Cailes et al., 2018). Among NICUs of the Vermont Oxford Network, three-quarters of infants given antibiotics for >48 h did not have any culture-proven bacterial infection (Ho et al., 2018).

Therefore, limiting uncontrolled antibiotics use in NW is currently one of the main objectives for all hospital teams as well as professional authorities, hospital administration, public health organizations, and researchers. Indeed, many studies, including a recent French survey (Leroux et al., 2015), indicate that antibiotics use varies widely between NICUs (Schulman and Saiman, 2011; Flannery and Puopolo, 2018). This variability has led to two essential questions from many authors about antibiotics use in NICUs: “how much is too much—or too little?” or “when to start and when to stop” (Schulman and Saiman, 2011; Bizzarro, 2018; Flannery and Puopolo, 2018; Dona et al., 2020). Answers are complex and do not simply rely on some strict universal measures for improving antibiotics use in NICU. Local conditions, such as microbiological flora characteristics, prenatal antibiotics exposure, and prevention of perinatal streptococcal group B infection, can also necessitate local adaptation of general guidelines for antibiotics prescription in NICUs. Therefore, a detailed analysis of each local situation could lead to tailored local actions and improved performance indicators.

We hypothesized that NICU performance could be rapidly improved if each NICU team is regularly given global and detailed information about its use of antibiotics in comparison to other NICUs. Therefore, the principal objective of this study was to describe changes in antibiotic exposure (AE) over 3 years in a large benchmarking network of NW in France where each center is regularly informed of its results, as well as the results of other centers, and each center has total autonomy to ameliorate its performance (Gouyon et al., 2019).

## Methods

### Study Design

This study was an exhaustive analysis of electronic prescriptions in a 23 level 3 neonatal wards (L3NWs) network. Prescriptions were automatically recorded prospectively and issued by a commercial computerized prescription order-entry system (CPOE) associated with a Clinical Decision Support (CDS) system (Logipren® software) between January 1st, 2017, and December 31st, 2019. Comprehensive data on antibiotic prescribing were provided once a year at a national user meeting where data from the centers and ways to improve were discussed. The main objective of the study was to analyze and evaluate annual changes in the AE rate. The secondary objective was to determine whether existing quantitative baseline measurements (days of therapy, DOTs) and a new qualitative baseline (antibiotic spectrum index, ASI) are useful.

### Neonatal Wards

Twenty-three L3NWs participated in a benchmarking program of neonatal medication prescribing practice (B-PEN program). Of these, 17 L3NWs participated since the beginning of the study and six joined in 2017. To evaluate the time-related changes in results over the three successive years, the hospitals collected the data for the three yearly key interventions (see the section below). Thirteen other hospitals joined the B-PEN program after 2017 and were not considered for this study.

### Characteristics of the CPOE/CDS System and Prospectively Recorded Data

The CPOE/CDS system (Logipren software; versions 1 and 2) and data recorded for each prescription have been previously described (Gouyon et al., 2017; Gouyon et al., 2019). In brief, this system allowed medication prescription according to indication, gestational age, postnatal age, postconceptional age, and body weight on the day of prescription. All electronic prescriptions were automatically stored in local computer servers. They were fully anonymized (deidentification) within each participating hospital before being sent to a common database for subsequent analysis. The authorization was given by the National Commission for Data Protection and Privacy (DE-2015-099 and DE-2017-410) and complied with the most recent French regulation MR-003, which governs research in the health field without the need of obtaining specific consent (CNIL, 2018).

### The Key Intervention

The key intervention consisted of an annual comparison of the benchmarking results by the B-PEN study group, which included pediatrician’s and neonatologist’s representative of each NICU. Parameters analyzed for population characteristics and antibiotics prescription were as follows: AE rate, international nonproprietary names (INNs), duration of exposure, and use of broad-spectrum antibiotics. Some information focused on the first three days of hospitalization: prescription of a triple combination of antibiotics and delayed end at the 4th day of antibiotics given for an unconfirmed infection. Each NICU staff received a complete report allowing comparison of its local performance to other deidentified centers. Report sending was followed by a meeting in the short term, allowing an analysis of changes from the previous meeting and variability in prescriptions between centers. Finally, each hospital had the choice of its specific actions, which were not recorded.

### Inclusion Criteria

All patients from L3NW with a first antibiotics prescription before their 28th day of life and with at least one electronic medication prescription were eligible for the study.

### Outcomes and Statistical Analysis

Only injectable and oral antibiotics prescriptions were considered in this study (see Supplementary Table S1). The antibiotic load was evaluated through quantitative and qualitative parameters. The primary parameter was AE. In addition, the following metrics were obtained: DOT and ASI. They were defined as follows (Public Health Ontario, 2019; CDC, 2021):

- AE was the number of neonates with at least one antibiotic prescription.
- DOT was the number of days the neonate was on a specific antibiotic, irrespective of the number of doses of that antibiotic received. If a single patient received two different INN antibiotics on 1 day, it was counted as two DOTs. DOT has been standardized to 1,000 patient-days by convention.
- ASI per antibiotic day was recently introduced in pediatric intensive care units and NICU (Gerber et al., 2017; Lahart et al., 2019). For ASI, each antibiotic received a score from 1 to 13 (1 for the most narrow-spectrum agent and 13 for the most broad-spectrum agent).

Results were presented using frequency and proportion for discontinuous variables and using the median and interquartile range (IQR) for continuous variables. The 95% confidence intervals (95% CI) were calculated for the main outcome. Cochran–Armitage tests for trend and Spearman’s rank correlation coefficient were applied. Statistical analysis was conducted using SAS® software (Version 9.4, SAS Institute, North Carolina, United States).

## Results

### Population Characteristics

**FIGURE 1:**
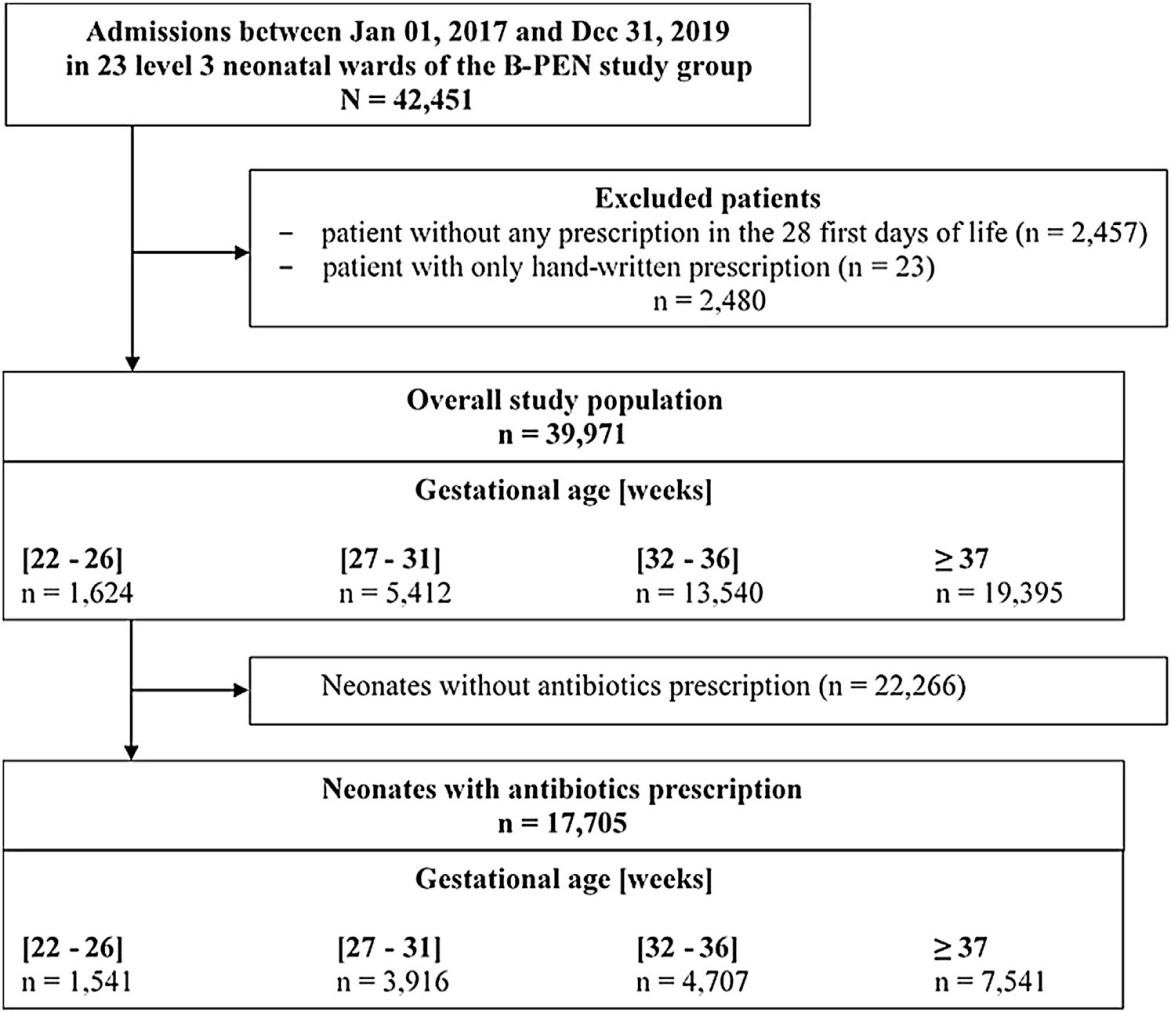
Flow chart of the study subjects.

Over the three years of the study, 42,451 neonates were hospitalized in the 23 L3NWs of the B-PEN network, and 39,971 of these neonates were included in this study (Figure 1). The main characteristics of the study population are shown in Table 1 overall and by gestational age (GA) groups. The incidence of preterm birth, defined as GA <37 weeks of gestation (WGs), was 51.5% in this overall population. The distribution of the study population by GA groups was 4.1% at 22–26 WGs (ELGA group), 13.5% at 27–31 WGs (VLGA group), 33.9% at 32–36 WGs, and 48.5% at term (≥37 WGs). In the overall population, 54.8% were males, the median value for their length of stay was 8 days, and the mortality rate was 2.9% (Table 1).

**TABLE 1:** Population characteristics and antibiotic exposure.

	Gestational age [weeks]				Overall study population, *n* = 39,971
	[22–26]	[27–31]	[32–36]	≥37	
	*n* = 1,624	*n* = 5,412	*n* = 13,540	*n* = 19,395	
Male, n (%)	838 (51.6)	2,913 (53.8)	7,285 (53.8)	10,851 (55.9)	21,887 (54.8)
Birth weight (g), median [IQR]	750 [650;860]	1,250 [1,015;1,480]	2,105 [1800;2,430]	3,210 [2,825;3,575]	2,480 [1780;3,210]
Length of stay (days), median [IQR]	60 [11;100]	40 [19;62]	12 [6;21]	4 [2;8]	8 [3;21]
Mortality at discharge, n (%)	419 (25.8)	257 (4.7)	177 (1.3)	297 (1.5)	1,150 (2.9)
Neonates by year of admission, n (%)					
2017	496 (30.5)	1,625 (30.0)	3,821 (28.2)	5,747 (29.6)	11,689 (29.2)
2018	558 (34.4)	1892 (35.0)	4,825 (35.6)	6,617 (34.1)	13,892 (34.8)
2019	570 (35.1)	1895 (35.0)	4,894 (36.1)	7,031 (36.3)	14,390 (36.0)
Cumulated number of patient-days, n	103,127	239,563	227,368	154,974	725,032
Antibiotics exposure, n (%)	1,541 (94.9)	3,916 (72.4)	4,707 (34.8)	7,541 (38.9)	17,705 (44.3)
[95% CI]	[93.8;96.0]	[71.2;73.5]	[34.0;35.6]	[38.2;39.6]	[43.8;44.8]
Male^a^, *n* (%)	796 (51.7)	2,198 (56.1)	2,794 (59.4)	4,519 (59.9)	10,307 (58.2)
Birth weight (g)^a^, median [IQR]	750 [653;859]	1,250 [1,010;1,485]	2,130 [1810;2,475]	3,305 [2,970;3,650]	2,330 [1,405;3,200]
Length of stay (days)^a^, median [IQR]	62 [12;102]	43 [20;66]	15 [7;28]	5 [3;10]	12 [4.0;35.0]
Mortality at discharge^a^, n (%)	397 (25.8)	225 (5.7)	137 (2.9)	204 (2.7)	963 (5.4)

^a^ Among neonates with antibiotic prescription.

CI, confidence interval; g, gram; IQR, interquartile range; n, number.

### Exposure to Antibiotics

At least one dose of antibiotics was prescribed to 17,705 neonates (44.3%; 95% CI: [43.8; 44.8]) over the total stay in the L3NW (Table 1). The lower the GA, the higher the AE rate: 94.9% in the ELGA group, 72.4% in the VLGA group, 34.8% at 32–36 WGs, and 38.9% in the at-term group.

Preterm infants were present at a higher proportion in the population with antibiotics prescription than in the overall study population (57.4% vs. 51.5%). Compared to the overall population, the antibiotic-treated subpopulation was shifted toward the ELGA group (8.7% vs. 4.1%) and the very low gestational age group (VLGA group; 22.1% vs. 13.5%), male gender (58.2% vs. 54.8%), long length of stay (12 vs. 8 days), and in-hospital death (5.4% vs. 2.9%).

Overall, 78.5% of the treated patients started their first antibiotic treatment in the first three days of life, 5.8% between the fourth and sixth day, and 15.7% after the sixth day (Table 2). A total of 46 different INN antibiotics were prescribed over the study period (see Supplementary Table S1 **)**. Gentamicin (73.4%), cefotaxime (58.1%), amoxicillin (ampicillin) (53.1%), vancomycin (23.3%), and amikacin (22.3%) were most often prescribed. The lower the GA was, the higher the exposure was to cefotaxime, vancomycin, and amikacin; however, the inverse was true for amoxicillin.

**TABLE 2:** Postnatal age at first prescription and top ten of INN antibiotics most prescribed.

	Gestational age [weeks]				Neonates with antibiotics prescription, *n* = 17,705
	[22–26]	[27–31]	[32–36]	≥37	
	*n* = 1,541	*n* = 3,916	*n* = 4,707	*n* = 7,541	
Postnatal age at first antibiotic prescription (days), *n* (%)					
D1	993 (64.4)	2,538 (64.8)	3,074 (65.3)	3,342 (44.3)	9,947 (56.2)
D2	154 (10.0)	359 (9.2)	681 (14.5)	1829 (24.3)	3,023 (17.1)
D3	51 (3.3)	141 (3.6)	166 (3.5)	566 (7.5)	924 (5.2)
D4	38 (2.5)	99 (2.5)	97 (2.1)	233 (3.1)	467 (2.6)
D5	32 (2.1)	81 (2.1)	73 (1.6)	147 (1.9)	333 (1.9)
D6	26 (1.7)	41 (1.0)	54 (1.1)	103 (1.4)	224 (1.3)
≥ D7	247 (16.0)	657 (16.8)	562 (11.9)	1,321 (17.5)	2,787 (15.7)
Neonates with INN antibiotics prescription, *n* (%)					
Gentamicin	1,187 (77.0)	2,845 (72.7)	3,451 (73.3)	5,517 (73.2)	13,000 (73.4)
Cefotaxime	1,340 (87.0)	3,163 (80.8)	2,721 (57.8)	3,058 (40.6)	10,282 (58.1)
Amoxicillin	483 (31.3)	1,173 (30.0)	2,498 (53.1)	5,256 (69.7)	9,410 (53.1)
Vancomycin	1,108 (71.9)	1,551 (39.6)	676 (14.4)	787 (10.4)	4,122 (23.3)
Amikacin sulfate	527 (34.2)	1,070 (27.3)	1,006 (21.4)	1,352 (17.9)	3,955 (22.3)
Metronidazole	355 (23.0)	575 (14.7)	376 (8.0)	303 (4.0)	1,609 (9.1)
Meropenem	201 (13.0)	190 (4.9)	64 (1.4)	54 (0.7)	509 (2.9)
Piperacillin-tazobactam	124 (8.0)	117 (3.0)	104 (2.2)	109 (1.4)	454 (2.6)
Cefepime	132 (8.6)	132 (3.4)	67 (1.4)	90 (1.2)	421 (2.4)
Amoxicillin-clavulanate	17 (1.1)	44 (1.1)	75 (1.6)	240 (3.2)	376 (2.1)

D, day; INNs, international nonproprietary names; n, number.

Gentamicin exposure rate significantly increased during the three successive study years in antibiotic-treated patients (from 70.1% to 74.1%; *p* < 0.0001) (see Supplementary Table S2). Prescription rate decreased for cefotaxime (62.5–54.6%; *p* < 0.0001 (Table 3)) and for amikacin (from 26.3% to 21.6%; *p* < 0.0001), while it remained unchanged for vancomycin and amoxicillin. In comparison to 2017, the rate of cefotaxime exposure declined by 7.8% in 2018 and by 12.6% in 2019. This decline is significant at 32–36 WGs (12.4% and 17.4%, respectively; *p* < 0.0001) and at term (13.3% and 20.3%, *p* < 0.0001) but not before 32 WGs (Table 3).

**TABLE 3:** Antibiotic exposure, combination therapies, and duration of therapy by year of admission of neonates.

	Gestational age [weeks]				Neonates with antibiotics prescription, *n* = 17,705
	[22–26]	[27–31]	[32–36]	≥37	
	*n* = 1,541	*n* = 3,916	*n* = 4,707	*n* = 7,541	
Antibiotics exposure by year of admission^a^, *n* (%)					
2017	479 (96.6)	1,212 (74.6)	1,462 (38.3)	2,318 (40.3)	5,471 (46.8)
2018	519 (93.0)	1,356 (71.7)	1,625 (33.7)	2,575 (38.9)	6,075 (43.7)
2019	543 (95.3)	1,348 (71.1)	1,620 (33.1)	2,648 (37.7)	6,159 (42.8)
*p*-value for trend	0.39	0.03	<0.0001	0.002	<0.0001
Number of INN antibiotics in combination at D1–D3^b^, *n* (%)					
Monotherapy	12 (1.0)	67 (2.2)	117 (3.0)	239 (4.2)	435 (3.1)
Double combination therapy	818 (68.3)	2,295 (75.5)	3,051 (77.8)	4,489 (78.2)	10,653 (76.7)
Triple combination therapy	368 (30.7)	676 (22.3)	753 (19.2)	1,009 (17.6)	2,806 (20.2)
Triple combination therapy prescribed at D1–D3^b^, *n* (%)					
2017	136 (36.2)	259 (26.8)	300 (24.7)	345 (19.5)	1,040 (24.0)
2018	120 (30.0)	236 (21.7)	256 (18.9)	335 (17.3)	947 (19.8)
2019	112 (26.5)	181 (18.4)	197 (14.6)	329 (16.2)	819 (17.1)
*p*-value for trend	0.003	<0.0001	<0.0001	0.008	<0.0001
Duration of the first treatment initiated at D1–D3^b^, *n* (%)					
<4 days	669 (55.8)	2,110 (69.5)	3,131 (79.9)	3,762 (65.6)	9,672 (69.6)
4 days	151 (12.6)	365 (12.0)	319 (8.1)	703 (12.3)	1,538 (11.1)
>4 days	378 (31.6)	563 (18.5)	471 (12.0)	1,272 (22.2)	2,684 (19.3)
Duration of the first treatment initiated at D1–D3 until the 4^th^ day^b^, *n* (%)					
2017	45 (12.0)	91 (9.4)	80 (6.6)	200 (11.3)	416 (9.6)
2018	52 (13.0)	150 (13.8)	119 (8.8)	231 (11.9)	552 (11.5)
2019	54 (12.8)	124 (12.6)	120 (8.9)	272 (13.4)	570 (11.9)
*p*-value for trend	0.73	0.03	0.04	0.05	0.0005
Neonates with cefotaxime prescription, *n* (%)					
2017	422 (88.1)	993 (81.9)	942 (64.4)	1,063 (45.9)	3,420 (62.5)
2018	457 (88.1)	1,101 (81.2)	917 (56.4)	1,026 (39.8)	3,501 (57.6)
2019	461 (84.9)	1,069 (79.3)	862 (53.2)	969 (36.6)	3,361 (54.6)
*p*-value for trend	0.12	0.09	<0.0001	<0.0001	<0.0001

^a^ Denominator is the number of neonates by year of admission.

^b^ Among neonates with antibiotic prescription initiated in the first 3 days of life.

D, day; n, number; INNs, international nonproprietary names.

**FIGURE 2:**
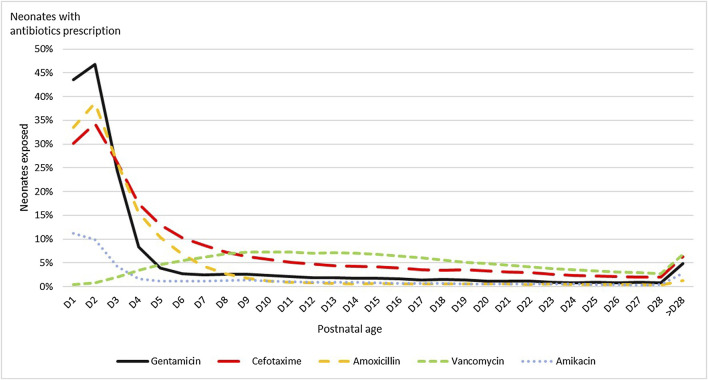
Neonates exposure by postnatal age to the five INN antibiotics most prescribed.

Cefotaxime, amoxicillin, and aminoglycosides were the antibiotics most often prescribed in the first three days of life (Figure 2). Then, vancomycin increased progressively and became the most prescribed antibiotic after day nine.

### Antibiotic Exposure Declined Over the Years

The AE rate significantly declined over the three successive years by 8.5% (46.8% in 2017, 43.7% in 2018, and 42.8% in 2019; *p* < 0.0001). This decrease varied according to GA, and the statistical significance was limited to the three groups with GA > 26 WGs: 1.3% at 22–26 WGs, 4.7% at 27–31 WGs, 13.6% at 32–36 WGs, and 6.5% at term (Table 3).

Since it is widely established that, in neonates, the first antibiotic course is usually initiated in the first three days of life, the evaluation committee of the program focused on specific targets of improvement in this early period. The incidence of prescription of a combination of three antibiotics as EONI treatment in the first three days after admission was 20.2%. This triple combination was 30.7% in ELGA and declined when GA increased (17.6% at term). It significantly decreased by 28.8% from 2017 to 2019 (*p* < 0.0001), and this decrease was significant in each GA group.

Among neonates treated in the first three days of life, 11.1% had the end of treatment on the fourth day of treatment. Those rates increased from 9.6% in 2017 to 11.9% in 2019 (*p* = 0.0005). This increase was significant for each GA group except ELGA.

### Prescriptions of Probiotics

Various probiotics were given the first week of life in ten hospitals to 932 neonates (2.3%) among the overall study population. At 22–26 WGs and 27–31 WGs, probiotics prescription rates were 4.9% and 6.7%, respectively (Supplementary Table S3 **)**.

### Metrics for Quantifying Antibiotics Use

Two metrics were used to quantify antibiotics use: DOT and ASI. DOT per 1,000 patient-days was 412.8 at 22–26 WGs, while it was markedly lower at 27–31 WGs (233.8) and at 32–36 WGs (162.1) (Table 4). The median ASI decreased significantly from 10.0 (IQR: 7.8; 11.6) at 22–26 WGs to 7.0 (IQR: 5.3; 8.5) at term (r = −0.32; *p* < 0.0001). This trend was found over the three years of the study (Table 4). The five most prescribed antibiotics (cefotaxime, gentamicin, amoxicillin, vancomycin, and amikacin) contributed to 92.1% of the ASI score in the treated neonates.

**TABLE 4:** Practice of antibiotics prescription by year of admission of neonates.

	Gestational age [weeks]				Neonates with antibiotics prescription, *n* = 17,705
	[22–26]	[27–31]	[32–36]	≥37	
	*n* = 1,541	*n* = 3,916	*n* = 4,707	*n* = 7,541	
DOT for 1,000 patient-days^a^	412.8	233.8	162.1	368.6	265.6
2017	398.6	232.5	172.4	370.4	271.3
2018	430.7	224.5	154.5	378.8	260.1
2019	490.8	244.1	161.6	357.7	266.0
ASI per antibiotic day, median [IQR]	10.0 [7.8;11.6]	9.0 [7.2;10.7]	8.0 [7.0;10.0]	7.0 [5.3;8.5]	7.9 [6.6;10.0]
2017	9.5 [7.6;11.0]	9.1 [7.2;10.8]	8.2 [7.0;10.0]	7.0 [5.3;9.0]	8.0 [6.7;10.0]
2018	10.0 [8.0;12.0]	9.0 [7.0;10.0]	8.0 [7.0;10.0]	7.0 [5.3;8.5]	7.7 [6.4;10.0]
2019	10.0 [7.9;11.5]	9.2 [7.5;11.0]	8.0 [7.0;10.0]	7.0 [5.3;8.3]	7.6 [6.7;10.0]
*p*-value for nullity Spearman’s ρ	0.09	0.08	<0.0001	0.03	0.002

ASI, antibiotic spectrum index; DOT, days of therapy; IQR, interquartile range; n, number.

^a^ Among overall study population.

## Discussion

This prospective observational study describes the evolution of antibiotic prescribing practices in 23 L3NWs, where a comprehensive electronic registry of all drug prescriptions allowed an in-depth comparative analysis of prescriptions by a steering committee representing each hospital. In addition, each team received its own results as well as those of the other L3NWs in anonymous form.

Some indicators decreased over the three years, namely, overall antibiotic exposure, exposure to cefotaxime, and the combination of three antibiotics to treat early neonatal infections. Conversely, stopping antibiotics on the fourth day of unnecessary treatment has increased from 9.6% to 11.9%. This study also shows that ELGA and VLGA neonates are in the worst position in terms of performance indicators and measurements.

Most pediatric interventions in hospital settings consist of audit and feedback interventions, followed by various types of guideline implementation (Dona et al., 2020). Two strong features of our benchmarking program are as follows:

- prescription information is recorded and stored automatically. Therefore, there is no need for any intervention of prescribers or research assistant.
- the B-PEN strategy is associated with an improvement in prescribing practice outside of any directive framework for corrective actions in NWs: each NW team received a comprehensive report of its own results and those of others; analysis of the steering committee was addressed to the teams who were entirely free to choose corrective actions without having to report on them. It should also be noted that the contribution of benchmarking to improving prescribing has been contemporaneous with national recommendations on early-onset neonatal infection in neonates over 33 weeks of gestational age. However, these French recommendations were known since 2016, published in 2017, and mainly concerned the diagnosis and treatment of neonates in maternity wards (de Santé, 2017).

The current study shows the delicate position of ELGA neonates in the domain of antibiotic therapy at birth. These ELGA neonates had the highest exposure rate (AE at 94.9%) and metrics of antibiotics use, while the lowest rates were in neonates whose GA exceeded 31 weeks (AE at 34.8% at 32–36 WGs). A recent study in 28 NICUs in the United States (Schulman et al., 2018; Flannery and Puopolo, 2018) also showed a substantial antibiotic load in ELBW neonates: the AE rate was 87.0% in the ELBW neonates and 78.6% in the very low birth weight neonates (1,000 ≤ BW < 1500 g) (Flannery and Puopolo, 2018). A similar AE rate of 84.9% was found in Canada, and a recent point prevalence survey study among 21 European countries also found the lowest gestational ages to be associated with the highest antibiotics exposure rates (Mesek et al., 2018). This study provides important information that shows that the benchmarking modalities implemented in this study did not benefit ELGA infants who did not experience any reduction in AE rates, except for a 26.8% reduction in the combination of three antibiotics. This decrease was, in any case, smaller than the reduction at 28–31 WGs (31.3%) and 32–36 WGs (40.9%).

Optimal management of the EONI risk in ELGA neonates is a complex issue, and limiting antibiotics prescription could be multifaceted and could benefit from more individualization of care, more precise information from maternal files about infectious and noninfectious causes of prematurity, optimization of laboratory tools for rapid diagnosis of EONI (bacteriological and biological), and preventive measures of late-onset neonatal infections.

The issue of cefotaxime treatment in NICUs provides an important example of the insights that can be gained from systematic and reliable data collection on drug treatment. Here, we show the preeminent position of cefotaxime, which ranks second among the 46 antibiotics prescribed. A recent large-scale study revealed that, in north-western Europe, the predominant antibiotic profile is gentamicin, ampicillin, benzylpenicillin, and vancomycin (Mesek et al., 2018). In contrast, in this study, it is gentamicin, cefotaxime, amoxicillin, and vancomycin. Moreover, this study reveals that the rate of cefotaxime prescription fell from 62.5% to 54.6%, and the decrease was predominant in the 32–36 WGs group.

Two severe microbiological conditions are considered the main drawback of the large-scale use of cefotaxime prescription in the NICU: *Escherichia coli* becoming resistant to cefotaxime, or even becoming multiresistant (Patel et al., 2009; Ho et al., 2018) and an increased risk of candidemia (Benjamin et al., 2014). Overall, the combination of three antibiotics in EONI treatment could contribute strongly to late nosocomial microbiological infection. Decreasing the triple antibiotics combination by 28.8% over the three years of the study is the first significant step toward a further decrease in cefotaxime prescription. Finally, it is worth noting that the rate of antibiotics discontinuation on the fourth day of treatment increased between 2017 and 2019, thus suggesting a low management of first treatment duration in uninfected–treated babies since a proven infection needs at least a 5–day treatment, while unconfirmed infection does not justify a treatment exceeding 2–3 days (Cailes et al., 2018).

Metrics of antibiotics use are parameters calculated for regional, national, and international comparisons in point prevalence surveys and antibiotic stewardship programs. DOT is regarded as a leading metric (Public Health Ontario, 2019; CDC, 2021). Our results rely on the continuous recording of all data regarding prescriptions. Overall values calculated for DOT and ASI were similar to those found in other studies (Schulman and Saiman, 2011; Gerber et al., 2017; Bizzarro, 2018; Schulman et al., 2018; Cailes et al., 2018). Moreover, all metrics of antibiotics use are inversely correlated to GA in this study.

The overall DOT per 1,000 patient-days sums all antibiotic days brought by each INN prescribed. However, DOT remained stable over the three years and did not identify the changes in AE rate, thus pointing to the need not to limit comparisons to global metrics but also to consider punctual targets issued from the practice in NWs (Lee et al., 2016; Nzegwu et al., 2017). With regard to the excessively long duration of antibiotic therapy, the SCOUT study (Cantey et al., 2016) recently showed that an automatic suspension of all antibiotics treatment at 48 h by a CPOE system forced prescribers to consider the benefit to stop or restart antibiotics. This sole quality method impacted the DOT, which was reduced by 27%.

Because the volume of prescribing and the spectrum of antibiotics were not correlated, two studies (Gerber et al., 2017; Lahart et al., 2019) proposed the ASI score as a complementary metric to add a new dimension in the evaluation of antibiotic stewardship program activities and their impact in NICUs (Gerber et al., 2017). Overall, the median ASI was highest in the ELGA group and showed a significant negative correlation with GA. ASI score also decreased over the 3 years of the study period in the groups with GA > 31 weeks. Since more than 90% of the ASI score in this study was related to the five most prescribed antibiotics, changes in ASI score with GA and years of study were a reflection of the cefotaxime prescription rate.

### Strengths and Weaknesses of the Study

The strength of this study lies in the large number of participating centers and newborns included, as well as its national representativeness of all gestational ages. In addition, the accuracy of the recorded data is due to the absence of any human intervention between prescription and storage. Users (mainly neonatologists and nurses) compare their data and possibly decide on local actions without any external control. Decided actions are not reported. This lack of information does not preclude our results, but other large and more comprehensive studies are needed to understand better the mechanisms of quality improvement in neonatal services and to evaluate the impact of the tool on the microbial ecology of NWs.

### Future

Practically, the best metrics and indicators for clinicians should have the strongest correlation with morbidity and mortality both in the short term and the long term in NWs.

## Conclusion

Continuous recording of all antibiotics prescriptions in NWs offers an opportunity for quality improvement in the same way as in pediatric and adult intensive care units. The advantages provided by these new opportunities largely overcome the disadvantages, and this study illustrates that practitioners in NWs have a key role to play and are ready to do so. Defining and creating optimal indicators as well as the best strategy to obtain them require further studies.

## Data Availability Statement

The raw data supporting the conclusions of this article will be made available by the authors, without undue reservation.

## Ethics Statement

The studies involving human participants were reviewed and approved by National Commission for Data Protection and Privacy, Commission Nationale de l’Informatique et des Libertés. Written informed consent from the participants’ legal guardian/next of kin was not required to participate in this study in accordance with the national legislation and the institutional requirements.

We thank the local coordinators responsible for the implementation of the benchmarking process, including actions issued from annual local results: Soumeth Abasse, CH Mayotte; Ceneric Alexandre, CHU Caen; Guillaume Binson, CHU Poitiers; Francesco Bonsante, CHU Sud Réunion; Roselyne Brat, CHR Orléans; Laurence Caeymaex, CHI Créteil; Marie-Laure Charkaluk, GHICL; Claire Claverie, CH Douai; Antonin Cornu, CH Metz-Thionville; Yvan Couringa, CH Cayenne; Cécile Desbruyeres, CH Chambery; Massimo Di Maio, CHU Nimes; Marine Dorsi, CHT Nouméa; Abdellah ElGellab, CH Lens; Guillaume Escourrou, CHI Montreuil; Florence Flamein, CHRU Lilles; Olivier Flechelles, CHU Martinique; Olivier Girard, CH St-Denis; Ghostine Ghida, CHU Amiens; Magloire Gnansounou, CHSA Maubeuge; Leila Karaoui, CH Meaux; Elsa Kermorvant-Duchemin, French Society of Neonatology; Yaovi Kugbe, CHOG; Catherine Lafon, CH Arras; Alexandre Lapillonne, Necker-Enfants Malades; Florence Le Bail Dantec, CH Saint-Brieuc; Laurence Martinat, CH Macon; Gaël Mazeiras, CH Cote-Basque; Julien Mourdie, CH Le Havre; Amélie Moussy-Durandy, CHI Poissy; Karine Norbert, CH Pau; Anne-Sophie Pages, CH Cotentin; Duksha Ramful, CHU Nord Réunion; Hasinirina Razafimahefa, CH Corbeil-Essonnes; Jean-Marc Rosenthal, CHU Guadeloupe; Magali Vidal, CH Perpignan; Amine Yangui, CHI Pontoise.

## Author Contributions

J-BG, SM-M, BG, and SI conceived the design and concepts. J-BG and SM-M wrote the manuscript. SL was in charge of the database and statistical analyses and contributed key information for Tables and Figures. All authors contributed to the article and approved the submitted version.

## Funding

This study is part of a European research program FEDER number 2014-34018 (https://ec.europa.eu/regional_policy/en/funding/erdf/) about benchmark of prescription in NICUs.

## Conflict of Interest

The authors declare that the research was conducted in the absence of any commercial or financial relationships that could be construed as a potential conflict of interest.

## References

[B1] Alexander V. N. Northrup V. Bizzarro M. J. (2011). Antibiotic exposure in the newborn intensive care unit and the risk of necrotizing enterocolitis. J. Pediatr. 159, 392–397. 10.1016/j.jpeds.2011.02.035 21489560 PMC3137655

[B2] Benjamin D. K. Jr. Hudak M. L. Duara S. Randolph D. A. Bidegain M. Mundakel G. T. (2014). Effect of fluconazole prophylaxis on candidiasis and mortality in premature infants: a randomized clinical trial. J. Am. Med. Assoc. 311, 1742–1749. 10.1001/jama.2014.2624 PMC4110724 24794367

[B3] Bizzarro M. J. (2018). Avoiding unnecessary antibiotic exposure in premature infants: understanding when (not) to start and when to stop. JAMA Netw. Open. 1, e180165 10.1001/jamanetworkopen.2018.0165 30646051

[B4] Cailes B. Kortsalioudaki C. Buttery J. Pattnayak S. Greenough A. Matthes J. (2018). Epidemiology of UK neonatal infections: the neonIN infection surveillance network. Arch. Dis. Child. Fetal Neonatal Ed. 103, F547–F553. 10.1136/archdischild-2017-313203 29208666

[B5] Cantey J. B. Wozniak P. S. Pruszynski J. E. Sánchez P. J. (2016). Reducing unnecessary antibiotic use in the neonatal intensive care unit (SCOUT): a prospective interrupted time-series study. Lancet Infect. Dis. 16, 1178–1184. 10.1016/S1473-3099(16)30205-5 27452782

[B6] CDC (2021). Antimicrobial use and resistance (AUR) module. Available at: https://www.cdc.gov/nhsn/PDFs/pscManual/11pscAURcurrent.pdf (Accessed December 24, 2020).

[B7] CNIL (2018). Commission Nationale de l’Informatique et des Libertés. Available at: https://www.cnil.fr/fr/declaration/mr-003-recherches-dans-le-domaine-de-la-sante-sans-recueil-du-consentement (Accessed December 24, 2020).

[B8] Cotten C. M. (2016). Adverse consequences of neonatal antibiotic exposure. Curr. Opin. Pediatr. 28, 141–149. 10.1097/MOP.0000000000000338 26886785 PMC4845665

[B9] Donà D. Barbieri E. Daverio M. Lundin R. Giaquinto C. Zaoutis T. (2020). Correction to: implementation and impact of pediatric antimicrobial stewardship programs: a systematic scoping review. Antimicrob. Resist. Infect. Contr. 9, 59 10.1186/s13756-019-0659-3 PMC7206826 32381059

[B10] Esaiassen E. Hjerde E. Cavanagh J. P. Pedersen T. Andresen J. H. Rettedal S. I. (2018). Effects of probiotic supplementation on the gut microbiota and antibiotic resistome development in preterm infants. Front. Pediatr. 6, 347 10.3389/fped.2018.00347 30505830 PMC6250747

[B11] Firestein M. R. Myers M. M. Austin J. Stark R. I. Barone J. L. Ludwig R. J. (2019). Perinatal antibiotics alter preterm infant EEG and neurobehavior in the Family Nurture Intervention trial. Dev. Psychobiol. 61, 661–669. 10.1002/dev.21820 30671945

[B12] Fjalstad J. W. Esaiassen E. Juvet L. K. van den Anker J. N. Klingenberg C. (2018). Antibiotic therapy in neonates and impact on gut microbiota and antibiotic resistance development: a systematic review. J. Antimicrob. Chemother. 73, 569–580. 10.1093/jac/dkx426 29182785

[B13] Flannery D. D. Puopolo K. M. (2018). Neonatal antibiotic use: how much is too much?. Pediatrics. 142 10.1542/peds.2018-1942 PMC6317556 30177518

[B14] Gerber J. S. Hersh A. L. Kronman M. P. Newland J. G. Ross R. K. Metjian T. A. (2017). Development and application of an antibiotic spectrum index for benchmarking antibiotic selection patterns across hospitals. Infect. Control Hosp. Epidemiol. 38, 993–997. 10.1017/ice.2017.94 28560946

[B15] Gouyon B. Iacobelli S. Saliba E. Quantin C. Pignolet A. Jacqz-Aigrain E. (2017). A computer prescribing order entry-clinical decision support system designed for neonatal care: results of the 'preselected prescription' concept at the bedside. J. Clin. Pharm. Therapeut. 42, 64–68. 10.1111/jcpt.12474 27882560

[B16] Gouyon B. Martin-Mons S. Iacobelli S. Razafimahefa H. Kermorvant-Duchemin E. Brat R. (2019). Characteristics of prescription in 29 Level 3 Neonatal Wards over a 2-year period (2017-2018). An inventory for future research. PLoS One. 14, e0222667 10.1371/journal.pone.0222667 31536560 PMC6752821

[B17] de Santé H. A. (2017). Prise en charge du nouveau-né à risque d’infection néonatale bactérienne précoce (>34 SA). Available at: https://www.sfpediatrie.com/sites/www.sfpediatrie.com/files/documents/label_has_recommandations_inbp.09.2017.pdf (Accessed October 13 2017).

[B18] Ho T. Buus-Frank M. E. Edwards E. M. Morrow K. A. Ferrelli K. Srinivasan A. (2018). Adherence of newborn-specific antibiotic stewardship programs to CDC recommendations. Pediatrics. 142, e20174322 10.1542/peds.2017-4322 30459258 PMC6589084

[B19] Lahart A. C. McPherson C. C. Gerber J. S. Warner B. B. Lee B. R. Newland J. G. (2019). Application of an antibiotic spectrum index in the neonatal intensive care unit. Infect. Control Hosp. Epidemiol. 40, 1181–1183. 10.1017/ice.2019.221 31352907

[B20] Lee K. R. Bagga B. Arnold S. R. (2016). Reduction of broad-spectrum antimicrobial use in a tertiary children’s hospital post antimicrobial stewardship program guideline implementation. Pediatr. Crit. Care Med. 17, 187–193. 10.1097/PCC.0000000000000615 26669645

[B21] Leroux S. Zhao W. Bétrémieux P. Pladys P. Saliba E. Jacqz-Aigrain E. (2015). Therapeutic guidelines for prescribing antibiotics in neonates should be evidence-based: a French national survey. Arch. Dis. Child. 100, 394–398. 10.1136/archdischild-2014-306873 25628457

[B22] Mesek I. Nellis G. Lass J. Metsvaht T. Varendi H. Visk H. (2018). Drug prescription pattern in European neonatal unitsBioRxiv. Available at: https://www.biorxiv.org/content/10.1101/463240v1.full.pdf (Accessed November 05 2018).

[B24] Nzegwu N. I. Rychalsky M. R. Nallu L. A. Song X. Deng Y. Natusch A. M. (2017). Implementation of an antimicrobial stewardship program in a neonatal intensive care unit. Infect. Control Hosp. Epidemiol. 38, 1137–1143. 10.1017/ice.2017.151 28745260

[B25] Patel S. J. Oshodi A. Prasad P. Delamora P. Larson E. Zaoutis T. (2009). Antibiotic use in neonatal intensive care units and adherence with Centers for Disease Control and Prevention 12 step campaign to prevent antimicrobial resistance. Pediatr. Infect. Dis. J. 28, 1047–1051. 10.1097/INF.0b013e3181b12484 19858773 PMC4526135

[B26] Public Health Ontario (2019). Antimicrobial stewardship programs (ASPs). Metrics examples. Available at: https://www.publichealthontario.ca/-/media/documents/A/2017/asp-metrics-examples.pdf?la=en (Accessed December 24, 2020).

[B27] Puopolo K. M. Mukhopadhyay S. Hansen N. I. Cotten C. M. Stoll B. J. Sanchez P. J. (2017). Identification of extremely premature infants at low risk for early-onset sepsis. Pediatrics. 140 10.1542/peds.2017-0925 PMC5654397 28982710

[B28] Schulman J. Saiman L. (2011). Metrics for NICU antibiotic use: which rate is right?. J. Perinatol. 31, 511–513. 10.1038/jp.2011.10 21796146

[B30] Schulman J. Profit J. Lee H. C. Dueñas G. Bennett M. V. Parucha J. (2018). Variations in neonatal antibiotic use. Pediatrics. 142 10.1542/peds.2018-0115 PMC6188671 30177514

[B32] Ting J. Y. Paquette V. Ng K. Lisonkova S. Hait V. Shivanada S. (2019). Reduction of inappropriate antimicrobial prescriptions in a tertiary neonatal intensive care unit after antimicrobial stewardship care bundle implementation. Pediatr. Infect. Dis. J. 38, 54–59. 10.1097/INF.0000000000002039 30531528

