# Supplementary material for: Antibiotics Prescription Over Three Years in a French Benchmarking Network of 23 Level 3 Neonatal Wards
Source: Front Pharmacol. 2021 Jan 25;11:585018. doi: 10.3389/fphar.2020.585018 (PMC7868323; doi:10.3389/fphar.2020.585018)
Supplement: Supplementary file 1 [file datasheet1.docx]

**Supplementary MATERIALS**

**Supplementary Table S1. List of INN antibiotics**

| Amikacin-sulfate | Ciprofloxacin | Ornidazole |
| --- | --- | --- |
| Amoxicillin | Clarithromycin | Oxacillin |
| Amoxicillin-clavulanate | Clindamycin | Penicillin |
| Azithromycin | Cloxacillin | Piperacillin |
| Cefaclor | Daptomycin | Piperacillin-tazobactam |
| Cefadroxil | Erythromycin | Pristinamycin |
| Cefamandole | Ethambutol | Pyrazinamide |
| Cefazolin | Fosfomycin | Rifampicin |
| Cefepime | Fusidic acid | Sulfadiazin |
| Cefixime | Gentamicin | Teicoplanin |
| Cefotaxime | Imipenem-cilastatin | Ticarcillin-clavulanate |
| Cefoxitin | Isoniazide | Tobramycin |
| Cefpodoxime | Josamycin | Trimethoprim-sulfamethoxazole |
| Ceftazidime | Linezolid | Vancomycin |
| Ceftriaxone | Meropenem |  |
| Cefuroxime | Metronidazole |  |

INN, International Non-proprietary Names

**Supplementary Table S2. Top five of INN antibiotics most prescribed by year of admission of neonates**

|  | **Gestational age [weeks]** | | | | **Neonates with** |
| --- | --- | --- | --- | --- | --- |
|  | **[22-26]** | **[27-31]** | **[32-36]** | **≥ 37** | **antibiotics prescription** |
|  | **n = 1541** | **n = 3916** | **n = 4707** | **n = 7541** | **n = 17705** |
| **Neonates with gentamicin prescription**, n (%) |  |  |  |  |  |
| 2017 | 369 (77.0) | 872 (71.9) | 1009 (69.0) | 1586 (68.4) | 3836 (70.1) |
| 2018 | 399 (76.9) | 1027 (75.7) | 1254 (77.2) | 1923 (74.7) | 4603 (75.8) |
| 2019 | 419 (77.2) | 946 (70.2) | 1188 (73.3) | 2008 (75.8) | 4561 (74.1) |
| p-value for trend | 0.96 | 0.27 | 0.01 | <0.0001 | <0.0001 |
| **Neonates with cefotaxime prescription**, n (%) |  |  |  |  |  |
| 2017 | 422 (88.1) | 993 (81.9) | 942 (64.4) | 1063 (45.9) | 3420 (62.5) |
| 2018 | 457 (88.1) | 1101 (81.2) | 917 (56.4) | 1026 (39.8) | 3501 (57.6) |
| 2019 | 461 (84.9) | 1069 (79.3) | 862 (53.2) | 969 (36.6) | 3361 (54.6) |
| p-value for trend | 0.12 | 0.09 | <0.0001 | <0.0001 | <0.0001 |
| **Neonates with amoxicillin prescription**, n (%) |  |  |  |  |  |
| 2017 | 175 (36.5) | 418 (34.5) | 760 (52.0) | 1544 (66.6) | 2897 (53.0) |
| 2018 | 161 (31.0) | 411 (30.3) | 876 (53.9) | 1809 (70.3) | 3257 (53.6) |
| 2019 | 147 (27.1) | 344 (25.5) | 862 (53.2) | 1903 (71.9) | 3256 (52.9) |
| p-value for trend | 0.001 | <0.0001 | 0.51 | <0.0001 | 0.90 |
| **Neonates with vancomycin prescription**, n (%) |  |  |  |  |  |
| 2017 | 347 (72.4) | 470 (38.8) | 197 (13.5) | 251 (10.8) | 1265 (23.1) |
| 2018 | 372 (71.7) | 506 (37.3) | 238 (14.6) | 265 (10.3) | 1381 (22.7) |
| 2019 | 389 (71.6) | 575 (42.7) | 241 (14.9) | 271 (10.2) | 1476 (24.0) |
| p-value for trend | 0.78 | 0.04 | 0.27 | 0.50 | 0.26 |
| **Neonates with amikacin-sulfate prescription**, n (%) |  |  |  |  |  |
| 2017 | 177 (37.0) | 347 (28.6) | 367 (25.1) | 550 (23.7) | 1441 (26.3) |
| 2018 | 172 (33.1) | 327 (24.1) | 283 (17.4) | 399 (15.5) | 1181 (19.4) |
| 2019 | 178 (32.8) | 396 (29.4) | 356 (22.0) | 403 (15.2) | 1333 (21.6) |
| p-value for trend | 0.17 | 0.60 | 0.05 | <0.0001 | <0.0001 |

INN, International Non-proprietary Names

n, number

**Supplementary Table S3. Exposure to probiotic agents in the 1st week of life**

|  | **Gestational age [weeks]** | | | | **Overall study** |
| --- | --- | --- | --- | --- | --- |
|  | **[22 - 26] n = 1624** | **[27 - 31] n = 5412** | **[32 - 36] n = 13540** | **≥ 37 n = 19395** | **population**  **n = 39971** |
| **Onset of probiotics in the first week of life**, n (%) | 79 (4.9) | 362 (6.7) | 372 (2.7) | 119 (0.6) | 932 (2.3) |
|  |  |  |  |  |  |
| **Onset of probiotics in the first week of life ***, n (%) | 69 (5.8) | 248 (8.2) | 169 (4.3) | 72 (1.3) | 558 (4.0) |

* among neonates with antibiotics prescription initiated in the first 3 days of life

n, number
